# Diagnosis and causal explanation in psychiatry

**DOI:** 10.1016/j.shpsc.2016.09.003

**Published:** 2016-12

**Authors:** Hane Htut Maung

**Affiliations:** Department of Politics, Philosophy, and Religion, Lancaster University, Lancaster, LA1 4YL, United Kingdom

**Keywords:** Psychiatry, Diagnosis, Causal explanation, Causal heterogeneity, Major depressive disorder

## Abstract

In clinical medicine, a diagnosis can offer an explanation of a patient's symptoms by specifying the pathology that is causing them. Diagnoses in psychiatry are also sometimes presented in clinical texts as if they pick out pathological processes that cause sets of symptoms. However, current evidence suggests the possibility that many diagnostic categories in psychiatry are highly causally heterogeneous. For example, major depressive disorder may not be associated with a single type of underlying pathological process, but with a range of different causal pathways, each involving complex interactions of various biological, psychological, and social factors. This paper explores the implications of causal heterogeneity for whether psychiatric diagnoses can be said to serve causal explanatory roles in clinical practice. I argue that while they may fall short of picking out a specific cause of the patient's symptoms, they can nonetheless supply different sorts of clinically relevant causal information. In particular, I suggest that some psychiatric diagnoses provide negative information that rules out certain causes, some provide approximate or disjunctive information about the range of possible causal processes, and some provide causal information about the relations between the symptoms themselves.

## Highlights

• Psychiatric diagnoses exhibit high degrees of causal heterogeneity and complexity.
• This suggests psychiatric diagnoses fall short of diagnostic ideal in medicine.
• However, some psychiatric diagnoses offer other sorts of explanatory information.

## 1 Introduction

When a patient presents to the clinic with a set of symptoms, one of the physician's tasks is to make a diagnosis that explains these symptoms. In somatic medicine, the diagnosis usually fulfils this explanatory role by indicating the cause of the patient's symptom presentation (Cournoyea and Kennedy, 2014, Schwartz and Elstein, 2008). For example, the diagnosis of myocardial infarction (MI) explains a patient's chest pain by indicating that the cause is ischaemic necrosis of the myocardium. This model of causal explanation suggests essentialism regarding disease kinds, whereby a diagnosis is taken to pick out a “disease entity” that can be a treated as a distinctive cause (Hucklenbroich, 2014). Moreover, this cause is taken to be invariant across cases, such as the diagnosis of MI referring to a causative pathology, ischaemic necrosis of the myocardium, which is instantiated by every case of MI.

The essentialistic thinking associated with this model of causal explanation continues to influence modern conceptions of psychiatric diagnoses (Haslam, 2014, Hyman, 2010). For example, the following passage from a psychiatric textbook characterises major depressive disorder (MDD) as a distinctive kind of disease that can cause the symptom of depression:

Depression is more common in older people than it is in the general population. Various studies have reported prevalence rates ranging from 25 to almost 50 percent, although the percentage of these cases that are caused by major depressive disorder is uncertain. (Sadock & Sadock, 2008, p.215)

A popular health information website does so similarly with generalised anxiety disorder (GAD):

GAD is a long-term condition that causes you to feel anxious about a wide range of situations and issues, rather than one specific event. (NHS Choices, 2016)

Similarly again, the following passage from a research paper on chronic fatigue syndrome (CFS) suggests that MDD refers to a distinctive disease that can explain fatigue symptoms:

When a well-recognized underlying condition, such as primary depression, could explain the subject's symptoms, s/he was classified as having “CFS-explained”. (Jason et al., 2014, p.43)

These sorts of characterisations are not surprising when we consider psychiatry's status as a medical discipline. As noted by Poland (2014, pp. 31–33), psychiatric practice occurs in a context shaped by medical roles and traditions. Hence, as in other medical disciplines, disorders in psychiatry are treated as distinctive disease kinds that can be invoked in causal explanations of patients' symptoms.

However, there are reasons to suspect that such essentialistic thinking may be misplaced in psychiatry. At present, it is unclear whether many of our current diagnostic categories actually do pick out stable and distinctive causes. Studies on the genetics and neurochemistry of psychiatric disorders have tended to indicate high degrees of heterogeneity, and while recent advances in cognitive neuroscience and functional neuroimaging have yielded compelling insights into the mechanisms involved in psychopathology, it is disputed whether they on their own could supply individual disease definitions (Hyman, 2010, p. 171). Therefore, we need to at least consider the possibility that many of the major psychiatric disorders could turn out to be causally heterogeneous at every level of analysis (Kendler, 2012, Murphy, 2006, Poland et al., 1994).

If it does turn out to be the case that psychiatric diagnoses cannot be understood essentialistically, then this would cast doubt on whether such diagnoses genuinely are of any causal explanatory value in clinical practice. This is not only an epistemological problem for philosophers, but is relevant to psychiatric researchers and practitioners. First, it calls into question the validity of our current diagnostic classification in psychiatry. That is, if it turns out that our current diagnostic categories do not represent distinctive disease kinds, then it is questionable whether they can be used to support inductive inferences and formulate laws (Cooper, 2005). Second, as argued by Haslam, the essentialisation of psychiatric disorders can encourage harmful stigma “because it represents sufferers as categorically abnormal, immutably afflicted, and essentially different” (Haslam, 2014, p. 25). Hence, if it turns out that diagnostic categories in psychiatry do not correspond to distinctive causal essences, then there is a possibility that such essentialistic conceptions of psychiatric disorders not only mislead patients, but also harm them.

In light of these concerns, it is worth asking whether there are other ways to think about the explanatory roles of diagnoses that do not encourage problematic reification. This will be the focus of this paper. I shall argue that even in the case that psychiatric diagnoses turn out to be causally heterogeneous at every level of analysis, they can still provide information that is explanatorily valuable in the clinical setting. Moreover, while these other forms of explanation do not fit the standard model of causal explanation whereby a diagnosis specifies a distinctive cause, I shall show that they are nonetheless causal in satisfying ways.

The paper proceeds as follows. I begin in Section 2 by distinguishing two types of explanatory question, which are the explanation of a syndrome in general and the explanation of the clinical presentation of a particular patient with appeal to a diagnosis. Using MDD as a case study, I explore the potential challenges that psychiatric disorders pose for these explanatory questions. While philosophers of psychiatry have offered promising approaches to the first kind of explanation that handle the challenges of heterogeneity and multilevel complexity, these problems continue to affect the second kind of explanation. Nonetheless, I argue in Section 3 that even though psychiatric diagnoses may turn out not to pick out homogeneous causal essences, there are other ways in which they might offer causal explanatory information. I suggest that some can provide negative information that excludes certain causes, some can provide partial explanations involving possible causal processes, and some can provide information about the causal relations between the symptoms themselves.

It should be made clear from the outset that my intention is not to argue that MDD definitely is a heterogeneous phenomenon. Rather, it is to explore the philosophical implications for the explanatory role of the diagnosis if it were to turn out to be causally heterogeneous. I use MDD as a case study, because it typifies a scenario where, given our current incomplete understanding of the causal processes involved, there remains a real possibility that there is no single causal essence that defines the disorder. However, even if it were to turn out that MDD is associated with a stable causal structure, there are other psychiatric diagnoses that are likely to be causally heterogeneous, and so my analysis of causal explanation would still be applicable. For example, some researchers suggest schizophrenia (Wheeler & Voineskos, 2014) and bipolar disorder (Maletic & Raison, 2014) might turn out to be causally heterogeneous.

## 2 Challenges for explanation in psychiatry

### 2.1 Two explanatory questions

Throughout this section, I use the example of MDD to highlight some of the challenges facing explanation in psychiatry. Before I turn to the case study, it is important to distinguish two kinds of explanatory question regarding diagnoses in medicine (Thagard, 1999, p. 20). The first kind, which I henceforth call “disease explanation”, belongs to medical research. This is the explanation of a clinical syndrome in general. The goal here is to develop a general model that brings together the relevant causal factors and mechanisms responsible for the syndrome. For example, the disorder characterised by swollen limbs and bleeding gums known as scurvy is explained by defective collagen synthesis due to ascorbic acid deficiency (Thagard, 1999, pp. 120–122). The second kind of explanation, which I henceforth call “diagnostic explanation”, occurs in the context of clinical practice. This is where a patient presents with such and such symptoms, and the physician makes a diagnosis that explains these symptoms. Take the example mentioned in Section 1 of patient's chest pain being explained by the diagnosis of MI. Here, the *explanandum* is not the clinical syndrome in general, but the clinical presentation of the particular patient.

These two explanatory questions are connected. In diagnostic explanation, where a diagnosis is invoked to explain a patient's symptoms, the understanding of the disorder picked out by the diagnosis comes from the general model that is constructed through disease explanation. For example, disease explanation informs us that MI in general involves rupture of an atherosclerotic plaque and thrombus formation leading to occlusion of a coronary artery and ischaemic necrosis of the myocardium, and it is in virtue of this knowledge that the diagnosis of MI functions as a causal explanation of the occurrence of chest pain in a particular patient. Hence, what the general model of a disorder looks like has implications for the explanatory function of the diagnosis in the particular case.

Much of the philosophical literature on explanation in psychiatry has focused on disease explanation, rather than diagnostic explanation. Theorists have expressed concerns that high degrees of heterogeneity and complexity could present significant challenges for developing comprehensive models of many major psychiatric disorders (Hyman, 2010, Murphy, 2006, Poland, 2014). However, as we shall see, this also has implications for diagnostic explanation. Note that it is not so much the heterogeneity of symptoms that is the problem, as many medical disorders that have been successfully modelled can present in several different ways. For example, syphilis has protean manifestations, which can include ulceration, gastric dysmotility, cardiac disease, and paresis, but these many different manifestations are unified by a singular cause, namely *Treponema pallidum* infection. Rather, the concerns are about the heterogeneity with respect to causes. Let us now turn to the case of MDD to illustrate these concerns.

### 2.2 Heterogeneity and complexity in major depressive disorder

Research on the genetics associated with MDD suggests that while genes increase vulnerability to MDD, environmental factors remain aetiologically more important.^1^ Several environmental factors have been established as being causally relevant, including poor parenting, sexual abuse, and stressful life events, but these vary across individuals (Kendler, Gardner, & Prescott, 2002). Regarding psychological factors, certain aspects of personality, such as neuroticism, have been correlated with vulnerability to MDD, but these are non-specific (Kendler, Gatz, Gardner, & Pedersen, 2006). And so, there is much evidence to suggest that the distal causes of MDD are highly heterogeneous.

However, it could be argued that heterogeneity with respect to such distal causes is not by itself problematic for disease explanation, because many medical disorders that have been successfully modelled have multiple risk factors. For example, distal causes for MI include genetic vulnerabilities, hypertension, obesity, smoking, and psychological stress, which vary significantly across cases. Nonetheless, these all converge onto a singular proximal cause, ischaemic necrosis of the myocardium, which is a defining feature of every case of MI. This suggests that we also need to look at whether the proximal causes of psychiatric disorders are heterogeneous. Two prominent neurochemical hypotheses regarding MDD are the monoamine hypothesis (Schildkraut, 1965) and the hypothalamic-pituitary-adrenal (HPA) axis hypothesis (Arborelius, Owens, Plotsky, & Nemeroff, 1999). These posit that depressive symptoms are caused by underactive serotonin (5-HT) neurotransmission and HPA axis dysregulation, respectively. While there is evidence that such neurochemical abnormalities are involved in many cases of MDD, research suggests that they are far from being universal.^2^ Therefore, they cannot be considered to be the defining essences of MDD.

Researchers have also investigated mechanisms at the level of brain circuitry. Using positron emission tomography (PET) and functional magnetic resonance imaging (fMRI) techniques, people with MDD were found to exhibit changes in the activation of the subgenual anterior cingulate cortex (ACC), which is an area of the brain that constitutes part of a circuit involved in emotional processing (Drevets et al., 1997, Groenewold et al., 2013, Mayberg et al., 1999). These results suggest that altered activity of the brain circuitry involved in emotional processing is associated with some of the affective symptoms of MDD. However, while significant, the findings are not universal. For example, a subsequent review by Drevets, Savitz, and Trimble (2008) notes that MDD patients who had first-degree relatives with mania, alcoholism, or sociopathy did not differ from healthy controls with respect to subgenual ACC metabolism or volume.^3^ Therefore, there still seems to be variability regarding the brain mechanisms among patients with the diagnosis of MDD.^4^

Another concern that further compounds the problem of heterogeneity is that it is contested whether conceptualising MDD exclusively at the level of brain mechanisms is sufficient for understanding some of the key features of its psychopathology. It is sometimes claimed that brain mechanisms are of utmost interest because they are the proximal causes of behaviour. However, there is no *a priori* reason to suppose that diseases must be defined by their most proximal causes. Furthermore, applying this neurocentrism universally can lead to trivial conclusions. For example, consider a patient presenting with a cough. Strictly speaking, the proximal cause of the cough *qua* behaviour is a neurological mechanism, namely the stimulation of the medulla by afferent fibres in the vagus nerve leading to the subsequent firing of efferent fibres that innervate the respiratory muscles. While this may be true, it is too trivial to be of explanatory significance in the clinic. Rather, we want a diagnosis to capture the causal process, albeit a less proximal one, that is perpetuating this neurological mechanism, such as a tumour, infection, inflammation, or cardiogenic pulmonary oedema, partly because this tells us where we can therapeutically intervene.

Similarly, in the case of a psychiatric disorder like MDD, the symptoms may indeed be mediated by proximal neurological mechanisms, but conceptualising the disorder exclusively at the level of these neurological mechanisms risks explanatory triviality, because it leaves out crucial information regarding the joint contribution of the other causal processes on which the maintenance of these neurological mechanisms is contingent and, moreover, on which it may be possible to intervene therapeutically. As with the cough example, it could be argued that an explanatory model of MDD would need to capture these processes to be of clinical value. Some theorists propose that such causal processes need not be restricted to internal physiological processes but could also include external social processes, on the grounds that these may be processes that are actively perpetuating the patient's condition (Broome and Bortolotti, 2009, Fuchs, 2012, Zachar and Kendler, 2007).^5^ And so, we need to take seriously the idea that psychiatric disorders can only be comprehensively understood as complex processes involving the interactions of causes across multiple levels (Kendler, 2012, Mitchell, 2008, Murphy, 2008).

The upshot of this subsection, then, is twofold. First, there is a possibility that MDD could turn out to be heterogeneous with respect to its proximal mechanisms and distal causes. Second, there are reasons to suppose that MDD cannot be aetiologically defined at any single level, but can only be understood by considering the causal processes that interact across levels. The following subsections look at the implications for disease explanation and diagnostic explanation in psychiatry.

### 2.3 Idealisation and pluralism in disease explanation

If concerns discussed in subsection 2.2 are genuine, then it would seem that a psychiatric disorder like MDD cannot be modelled essentialistically. The question, then, is how we can possibly construct a general model of the disorder given the problems posed by causal heterogeneity and multilevel complexity. Theorists have proposed idealisation (Murphy, 2006) and explanatory pluralism (Kendler, 2012, Mitchell, 2008) as solutions to these two problems, respectively.

In response to the problem of heterogeneity, Murphy (2006) suggests what when we try to explain a syndrome in general, what we are aiming to explain is an exemplar, which is an idealised theoretical representation of the syndrome. An exemplar *qua* idealisation is abstracted away from the idiosyncrasies of individual patients. Given the high degree of variability between cases of a given diagnosis, different patients may resemble the exemplar in different respects and to varying degrees. According to Murphy, to explain a syndrome is to model the various causal relations and mechanisms that have been shown to contribute to the development of the idealised syndrome described in the exemplar. Again, such a model represents an idealised scenario, abstracted away from the actual states of affair in particular cases. There may not be a single causal factor in the model that is instantiated by every case of the disorder and there may not be an actual case that instantiates all of the causal relations described in the model.

In response to the problem of multilevel complexity, Mitchell (2008) endorses a view called integrative pluralism, according to which a satisfactory explanation of a complex system like a psychiatric disorder requires the integration of causal components at multiple levels of organisation. As noted by Murphy (2008), these variables at different levels do not correspond to the same phenomenon described in different ways, but correspond respectively to different phenomena. Hence, it is insufficient to look for deterministic regularities exclusively at a single level, because whatever influence the variables at this level may have is heavily contingent on the joint contribution of variables at other levels. In the case of MDD, we might need to include information about genetic susceptibilities, neurochemical abnormalities, brain circuits, psychological vulnerabilities, social contextual factors, and the ways in which these interact.

Similarly, Kendler (2012) endorses an empirically-based pluralism. Given the diverse range of causal variables involved, he argues that there is no single privileged level at which a psychiatric disorder like MDD can be aetiologically defined. Rather, constructing a general model of the disorder requires the incorporation of research from different disciplines. Kendler (2014) suggests two philosophical approaches to causation that can guide this project. The first is Woodward's (2003) interventionist theory of causation, which conceptualises causal factors as difference makers, without placing ontological restrictions on the kinds of variable that can be such difference makers. This allows the inclusion of different causal factors regardless of the explanatory levels to which they belong. The second is the mechanistic approach to causation advocated by theorists such as Machamer, Darden, and Craver (2000), which focuses on specifying the mechanisms via which the identified difference makers interact to produce the clinical features of the disorder.

And so, with respect to disease explanation in psychiatry, there is recognition among contemporary theorists in the philosophy of psychiatry that general explanatory models of disorders are idealisations abstracted away from the heterogeneity of actual cases and that they involve the integration of diverse kinds of causal variable from different levels of organisation. However, significantly less has been written in the philosophical literature about diagnostic explanation in psychiatry. This is the focus of what is to follow.

### 2.4 Problems for diagnostic explanation

Idealisation and explanatory pluralism are promising strategies for disease explanation in psychiatry. When we want to understand what causes depressive symptoms in general, we can conjure up an idealised general representation of the syndrome and model the causal factors that are known to contribute to the phenomena described in the representation. The resulting model can then illuminate statistical generalisations and causal regularities at a population level, in spite of the heterogeneity seen in individual cases. However, I argue that heterogeneity remains problematic for diagnostic explanation when we try to apply the model to the individual cases.

As noted in subsection 2.2, there is a possibility that a psychiatric disorder like MDD does not involve a single invariant pathology that is instantiated in every case, but a vast range of diverse factors, none of which is universally present across all cases. Hence, as noted by Murphy, the same set of symptoms may be produced by different sets of causes:

It seems unlikely that the same underlying causes explain an irritable adolescent who sleeps late, diets frantically, and lies around the house all day threatening to commit suicide on the one hand, and a sad middle-aged man who can not settle down to any of his normal hobbies, hardly sleeps, eats more and more, can not make love to his wife, and feels worthless.^6^ (Murphy, 2006, p.329)

Similarly, Mitchell (2008, p. 30) suggests that there may be different routes leading to the same symptoms in different individuals. A general model of MDD, then, would need to represent the multiple causal pathways that could be responsible for the development of depressive symptoms.

This has implications for what sort of causal information the diagnosis of MDD conveys when a patient who presents to the clinic with mood symptoms is given the diagnosis. It would suggest that the diagnosis does not unequivocally specify a distinct “disease entity” that is responsible for the patient's symptoms in the particular case. Rather, it subsumes a range of possible causal structures that could be instantiated by the patient. Another way to interpret this is to say that MDD is a disjunctive category. Take *C*_1_, *C*_2_ … *C*_*n*_ to be the diverse causal variables that have been implicated in its pathophysiology. These may interact in different combinations to produce different underlying pathological states, *P*_1_ = {*C*_1_ … *C*_*x*_}, *P*_2_ = {*C*_2_ … *C*_*y*_}, … *P*_*n*_ = {*C*_*n*_ … *C*_*z*_}, each of which can produce the clinical syndrome that satisfies the diagnosis of MDD. Diagnosing a particular patient with MDD, then, indicates that the underlying state responsible for the patient's symptoms could be *P*_1_ or *P*_2_ … *P*_*n*_, but does not provide further causal discrimination beyond this.

Furthermore, different cases of MDD may need to be understood with different theoretical frameworks. As noted in subsection 2.3, the problem of multilevel complexity suggests that a general model of the disorder needs to integrate different kinds of causal variable (Kendler, 2012, Mitchell, 2008). With respect to individual cases, it is possible that the different combinations of variables instantiated by different patients with MDD may require different explanatory perspectives. For example, cognitive, psychodynamic, and social explanatory perspectives may be of more value for a patient with psychosocial adversity and a history of childhood trauma, while more emphasis may be placed on a neurobiological explanatory perspective for a patient with late-onset depression characterised by melancholic features.

This further supports the contention made by Poland et al. (1994) that a psychiatric diagnosis like MDD lacks unity. Not only can different patients diagnosed with MDD instantiate different underlying causal structures, but these different causal structures may need to be understood with appeal to different theoretical frameworks. Poland (2014) argues that this lack of unifying invariance makes the diagnostic categories in psychiatry poor tools for clinical practice. He suggests that a psychiatric diagnosis does not effectively contribute to serving important clinical functions because it “leaves most of the important clinical assessment work undone” (Poland, 2014, p. 35). By subsuming different patients with diverse pathologies under the same category, a diagnosis masks information about individual variation that could be important for treatment selection and prognosis. For example, the undifferentiated diagnosis of MDD does not discriminate between the patient with a dramatic onset of melancholic symptoms, for whom a tricyclic antidepressant and electroconvulsive therapy may be warranted, and the patient with a history of trauma, for whom psychotherapy may be more appropriate. Both patients would be subsumed under the same category of MDD.

The above criticism paints a rather pessimistic picture of psychiatric diagnoses. However, while I agree that the above mentioned problems significantly impact the clinical roles of diagnoses in psychiatry, I do not go as far as to say that the diagnoses contribute little or nothing of epistemic value to the clinical process. In section 3, I argue that while psychiatric diagnoses may not pick out specific causes, there are still ways in which they supply causal information that is of explanatory value.

## 3 Other kinds of diagnostic explanation in psychiatry

### 3.1 Negative causal explanation

One sort of causal information that can be provided by a psychiatric diagnosis is negative causal information. While a psychiatric diagnosis may not specify the precise causal process leading to the patient's symptom presentation, it nonetheless excludes certain causes. To better understand how this works in clinical practice, we need to look at the process of differential diagnosis, which is where the physician considers multiple possible diagnoses that could explain the patient's symptoms before selecting the diagnosis that best explains them. For example, after assessing a patient with chest pain, a physician may consider gastro-oesophageal reflux disease, pulmonary embolism, and MI as possible causes, before inferring that MI is the correct diagnosis.

For MDD, other conditions to be considered in the differential diagnosis include thyroid disorders, adrenal disorders, dementia, cerebral tumours, nutritional deficiencies, drug or alcohol intoxication, and other psychiatric disorders. When assessing a patient with depressive symptoms, it is recommended that he or she is appropriately investigated for these conditions:

The workup should include tests for thyroid and adrenal functions because disorders of both of these endocrine systems can appear as depressive disorders. In substance-induced mood disorder, a reasonable rule of thumb is that any drug a depressed patient is taking should be considered a potential factor in the mood disorder.^7^ (Sadock & Sadock, 2008, p. 217).

In the fifth edition of the *Diagnostic and Statistical Manual of Mental Disorders*, it is recommended that MDD should only be diagnosed once these other medical diagnoses have been excluded:

Such symptoms count towards a major depressive diagnosis except when they are clearly and fully attributable to a general medical condition. (American Psychiatric Association, 2013, p.164)

What this highlights is a the diagnosis of MDD is not made solely on the basis of the relevant symptoms being present, but also requires certain medical causes for the symptoms to be ruled out. A patient who presents with depressive symptoms that turn out to be caused by a cerebral tumour would not be diagnosed with MDD, because the diagnosis is excluded by the fact that the symptoms are clearly and fully attributable to a general medical condition. In virtue of this exclusion criterion, then, a psychiatric diagnosis provides information about what is not in the causal history of the patient's clinical presentation. A diagnosis of MDD may not pick out a specific cause of the patient's mood symptoms, but it does suggest that they are not being caused by hypothyroidism, drug intoxication, a tumour, and so on.

Lewis (1986) argues that this exclusion of causes still qualifies as a legitimate sort of causal explanation. According to his account of causal explanation, “to explain an event is to provide some information about its causal history” (Lewis, 1986, p. 217). This does not necessarily entail specifying a cause of the event, as there are other kinds of information one can give about an event's causal history, including information about what is not in its causal history. For Lewis (1986, p. 222), negative causal information can still be explanatorily relevant information, and so a psychiatric diagnosis can be explanatorily relevant by excluding certain causes, even if it does not itself cite a specific cause.

Beebee (2004) offers a modal analysis of how negative causal information can be explanatorily relevant. She argues that information about the absence of an event provides information about the causal processes in counterfactual worlds where that event occurs. For example, consider that Flora normally waters the orchids regularly, but forgets on one occasion. According to Beebee, Flora's failure to water the orchids cannot be a cause, because it does not denote an event, but rather the absence of an event. Nonetheless, we still accept Flora's failure to water the orchids as an explanation of the orchids dying. This is because it provides information about the causal histories of the nearby possible worlds where Flora had not failed to water the orchids and how these causal histories differ from the causal history of the actual world. In these counterfactual worlds, the causal processes would have ensued in such a way that the orchids would have survived.

It is worth acknowledging that Beebee's analysis focuses specifically on the roles of absences in causal explanations, and so is not wholly analogous with my example of diagnostic explanation in psychiatry. Nonetheless, it highlights the general point that a causal explanation does not have to cite a specific cause, but can provide modal information about the possible causal histories of the *explanandum*. I suggest that a similar modal analysis can be applied to other cases of negative causal information, including diagnoses in psychiatry. By indicating that the patient's mood symptoms are not attributable a general medical disorder, the diagnosis of MDD is providing information about what would have been expected in the counterfactual worlds where the patient's mood symptoms are attributable to a general medical disorder. For example, in the actual world, the physician might only diagnose a patient with MDD after a thyroid function test yields a normal result, which suggests that the result from the thyroid function test would have been abnormal in the possible world where the patient is not diagnosed with MDD due to his or her mood symptoms being attributable to a thyroid disorder.

This negative causal explanation can be valuable in the clinical setting. First, it has utility in predicting outcomes and guiding therapeutic interventions. Indicating that a patient's mood symptoms are not due to hypothyroidism suggests that levothyroxine supplementation would not be a therapeutically effective intervention and indicating that they are not due to a cerebral tumour suggests that neurosurgical referral is not required. Hence, by excluding these causes, a diagnosis of MDD can inform clinical decisions. Second, even if it does not specify precisely what is causing his or her symptoms, a psychiatric diagnosis can offer relief and reassurance by ruling out certain medical diagnoses. For example, when the family of a patient with a new onset of anhedonia, poor concentration, and psychomotor retardation want to know why the patient has these symptoms, they may find it extremely valuable to know that the symptoms are not caused by a cerebral tumour or a neurodegenerative disease.^8^

This account of negative causal explanation, then, suggests that a psychiatric diagnosis does not need to identify a specific disease kind to be of causal explanatory value, but can be explanatorily valuable in virtue of the exclusion criterion that states that the symptoms must not be attributable to a general medical disorder. As noted by Beebee (2004), “*E* because *C*” is not equivalent to “*C* causes *E*”. Hence, the causal claim “the patient's mood symptoms are caused by MDD” may indeed be misguided, we can still legitimately make the explanatory claim “the patient has mood symptoms because of MDD”.

However, in spite of the usefulness of negative information in the clinical setting, the account of diagnostic explanation presented here has limits. One problem is that it sets the standard for an acceptable causal explanation too low. If all that is needed for a causal explanation is information about what is not the cause of the *explanandum*, then all sorts of claims that we would not normally consider to be explanations would qualify as causal explanations. For instance, “not tuberculosis” would count as a causal explanation of a patient's chronic cough according to the negative causal explanation account. In response, one could propose that the strength of a negative causal explanation depends on how many causal possibilities are excluded by the *explanans*. Hence, MDD is a better explanation than “not tuberculosis”, because the former excludes several medical disorders while the latter only excludes tuberculosis. Nonetheless, this would still relegate psychiatric diagnoses to the same controversial status as the so-called medically unexplained syndromes, which also exclude several medical disorders as causes of patients' symptoms yet are widely considered to be explanatorily unsatisfactory (Cournoyea & Kennedy, 2014).

Another problem is that in practice there are many instances where psychiatric diagnoses are made without other medical disorders being excluded. In the above discussion, I have been considering an idealised case of differential diagnosis where a patient presents with a new onset of mood symptoms and different diagnoses are presented as possible explanations of the mood symptoms. Here, the diagnoses of MDD, hypothyroidism, and drug intoxication are presented as competing hypotheses, and the diagnosis of MDD is only established when the other diagnoses have been adequately excluded. However, there are also cases where a psychiatric disorder is not considered as a competing diagnosis, but as a comorbid diagnosis. For example, MDD is often treated as an additional comorbid diagnosis in patients with multiple sclerosis (MS), even though it is recognised that in these cases the depressive symptoms may be caused by the pathology associated with the MS (Marrie et al., 2009). Hence, in this sort of scenario, the diagnoses of MDD fails to exclude MS from the causal history of the patient's mood symptoms.

And so, while psychiatric diagnoses do sometimes provide valuable negative causal explanations, it is implausible that their entire explanatory worth lies only in their providing negative information. In the following subsections, I argue that they can also provide positive causal information. While these sorts of positive causal information fall short of picking out the specific causative pathologies in individual cases, they may nonetheless be explanatorily valuable in the clinical context.

### 3.2 Disjunctive causal explanation

The claim that psychiatric diagnoses do provide some positive causal information about patients' symptoms is corroborated by the fact that we have at least some scientific knowledge of the causal factors associated with certain disorders. As noted in subsection 2.3, even though there is high heterogeneity among cases MDD, we can seek to understand MDD in general by constructing an idealised model that is abstracted away from the idiosyncrasies of individual cases. Murphy (2014) argues that although patients may differ from the idealisation in different respects and to different degrees, the model can nonetheless provide at least an approximation of the causal processes in the individual case:

The bet is that real patients will be similar to the exemplar in enough respects so that the explanation of the exemplar carries over to the patient. We assume that within the individual there are phenomena and causal relations that are relevantly similar to those worked out for the exemplar, but we cannot expect very precise predictions. (Murphy, 2014, p.106)

The suggestion here is that while a psychiatric diagnosis *qua* idealised generalisation may not specify the precise causal structure underlying the patient's symptoms in a particular case, it does tell us about processes that are approximately similar to the actual causal processes in the patient's case. Hence, Murphy argues that a psychiatric diagnosis is explanatorily significant, because it gives us at least a vague idea of the sort of process that is producing the patient's symptoms.

However, I argue that things are more complicated than this. While the above picture acknowledges the high degree of variation between individuals, it rests on the assumption that cases of the disorder nonetheless share a similar sort of causal process (Murphy, 2014, p. 106). As noted in subsection 2.2, though, it is possible that there are different sets of causes leading to the same symptoms in different individuals, and so a general model of the disorder would need represent the different routes via which the syndrome can be produced. For example, it is possible that depressive symptoms are not caused by a single invariant pathology, but may be associated with a disjunction of several underlying states, *P*_1_ or *P*_2_ … *P*_*n*_, each produced by a different combination of interacting causal variables.

This might be viewed as problematic, because it is a matter of contention whether or not such disjunctive information can constitute an explanation. According to Kim (1998), it cannot. He argues that information about a disjunction of possible causes does not yield a single explanation with a disjunctive cause, but a disjunction of different possible explanations of which the correct explanation remains unknown. His example is the symptom of joint pain, which can be caused by a number of different disorders, including rheumatoid arthritis (RA) and systemic lupus erythematosus (SLE). Consider that a patient with joint pain undergoes a clinical test, the result of which suggests that he or she either has RA or SLE, but does not indicate which. Kim argues that we do not yet have an explanation of the patient's joint pain:

I think there is a perfectly clear and intelligible sense in which we don't as yet have an explanation: what we have is a disjunction of two explanations, not a single disjunctive explanation. What I mean is this: we have two possible explanations, and we know that one or the other is the correct one but not which it is. What we have, I claim, is not an explanation with a “disjunctive cause”, having rheumatoid arthritis or lupus. There are no such “disjunctive diseases”. (Kim, 1998, p.108)

Kim further qualifies this by arguing that “RA or SLE” *qua* disjunction does not specify a kind of event, and so is not eligible as a cause. Because it is not eligible as a cause, it cannot then be “citable as a *cause in a causal explanation*” (Kim, 1998, p. 109).

If Kim is right, then there is reason to suppose that MDD does not offer a positive causal explanation of a patient's mood symptoms, because it is associated with a range of many possible underlying causal structures but does not specify which one is actually the case in the patient. However, Kim's criteria for explanation are too restrictive. Even if a disjunctive category does not meet the explanatory ideal of picking out a specific cause, I argue that it can nonetheless provide some causal explanatory information. As noted in subsection 3.1, it is not necessary to cite a specific cause of an *explanandum* in order to provide explanatorily relevant information about the *explanandum*'s causal history. For example, one could give information about the possible causal histories within which the *explanandum*'s actual causal history lies. I suggest that this is the sort of information a disjunctive category provides.

We can highlight the explanatory relevance of a disjunctive diagnosis by reframing the language in Kim's example. Suppose we say that the test result indicates that the patient has a multisystem autoimmune disease. This is a heterogeneous category that includes RA and SLE. Hence, stating that the patient has a multisystem autoimmune disease is equivalent to stating that that he or she has the disjunction “RA or SLE …” without specifying which of these disorders he or she actually has. Nonetheless, it is generally agreed that indicating that the patient has a multisystem autoimmune disease is still explanatorily relevant with respect to his or her joint pain (Rose & Mackay, 1985). Not only does it greatly narrow down the range of conditions in which the patient's actual condition could lie, but it also provides positive causal information about the conditions that do fall within this range. The diagnosis of multisystem autoimmune disease tells us that the patient's joint pain could be caused by the erosion of the joint surfaces in the case of RA, or by systemic inflammation of the connective tissues in the case of SLE, and so on.

A disjunctive diagnosis, then, does not specify the actual cause of the patient's symptoms, but it nonetheless subsumes the actual cause within a tighter range of possible causal histories than otherwise would have been available and, moreover, provides some indication of the mechanisms involved in these possible causal histories. This information indicates differences between the causal histories of patients with the diagnosis and those of patients without the diagnosis that can inform further investigations and therapeutic interventions. For instance, stating that a patient has a multisystem autoimmune disease suggests that his or her condition is likely to respond to treatments that act on the immune system and provides a rational basis for further investigations, such as blood tests for specific autoantibodies, which can help specify whether he or she actually has RA or SLE.^9^

The above analysis accommodates the notion that a psychiatric diagnosis *qua* disjunctive category could still provide explanatorily valuable information about a patient's symptoms, even if it does not specify the precise cause of these symptoms. The diagnosis of MDD, for instance, might be taken to suggest that the patient's symptoms could be due to a state involving underactive 5-HT neurotransmission plus variables *C*_1_ … *C*_*n*_, or by a state involving HPA dysregulation plus variables *C*_2_ … *C*_*n*+1_, and so on. The explanatory value of this disjunctive information is that it tells us some of the ways in which the possible causal structures that could be underlying the patient's symptoms might differ from the causal structure of the non-depressed state. In this sense, the explanatory role of a psychiatric diagnosis like MDD may be more akin to that of a superordinate category like multisystem autoimmune disorder than to that of a specific medical diagnosis like RA.

However, while this analysis shows that disjunctiveness does not necessarily preclude a diagnosis from being explanatory, it could be argued that this explanatory value is also contingent on other conditions. First, it is contingent on whether an exhaustive list of disjuncts can be specified. The superordinate category of cancer is explanatorily valuable, because we are able to specify the different kinds of malignancy that fall under the category and have impressive knowledge of their respective causal structures. By contrast, we are far from being able to specify all the possible causal structures that fall under the category of MDD, or indeed say how many there are. As noted in subsection 2.2, we know a number of the causal variables that can be associated with MDD, but we still know very little about how different combinations of these variables interact to produce symptoms in individual cases.

Second, even if some of the disjuncts included in the category could be specified, one might argue that the explanatory value of the category is still contingent on whether we are capable of finding out precisely which disjunct is involved in any given case. This might be made possible with the discovery of biomarkers which indicate specific causal factors that may be potential targets for intervention. Recent neuroscientific data has suggested some potential avenues for biomarker research, such as the review of fMRI and PET studies by Roiser, Elliott, and Sahakian (2012), which found differential responses to pharmacological treatment and psychological therapy for patients with and without abnormal ACC activity. However, with respect to biomarker tests that could be readily used in clinical practice, rather than just in the research laboratory, psychiatry has fallen short of other medical specialties. Hence, we may currently be in a situation where research can discover various causal factors associated with a psychiatric disorder, but we cannot match them to individual patients in the clinic.

As a modest response to the above two concerns, a disjunctive category could still provide causal explanatory information of a statistical nature regarding the patient's condition. Even if we cannot specify all of the possible disjuncts that fall under the category or find out which disjunct is involved in any given case, the diagnosis still indicates an increased probability of the patient having a given causal mechanism. For example, on the basis of the knowledge that a proportion of people with MDD have underactive 5-HT neurotransmission, we can say that a given patient with a diagnosis of MDD has an increased probability of having underactive 5-HT neurotransmission. This might provide some justification for a trial of antidepressant medication, which is presumed to exert its action via 5-HT. However, it also needs to be acknowledged that many similar probabilistic causal explanations in psychiatry may be of little clinical value, because the diagnosis might turn out to be too causally heterogeneous to have explanatory significance. The number of disjuncts in the category may be so vast that there is only a minute statistical association between each causal variable and the disorder. Therefore, knowledge of such causal variables may, on average, be statistically unhelpful in the guiding of treatment.

In summary, a disjunctive analysis accommodates the possibility of a heterogeneous diagnostic category being of causal explanatory value. However, this explanatory value is also dependent on other considerations, including the extent of heterogeneity, whether the disjuncts can be exhaustively specified, and whether we are able to find out which causal variables are involved in any given case. Given the ongoing challenges for research into causal pathways and biomarkers in psychiatry, it must be conceded that at present the positive causal explanatory value of a psychiatric diagnosis *qua* disjunctive category is modest at best.

### 3.3 Causal networks of symptoms

The third sort of causal information a psychiatric diagnosis can provide is information about the causal relations that occur between the symptoms and sustain them as a stable cluster. This draws on a recent development in the study of psychopathology, namely the symptom network approach to psychiatric disorders advocated by Borsboom (2008) and Cramer, Waldorp, van der Maas, and Borsboom (2010). According to this approach, a psychiatric disorder is conceptualised as a network of symptoms that reinforce each other via causal relations. For example, in the case of MDD, “fatigue may lead to a lack of concentration, which may lead to thoughts of inferiority and worry, which may in turn lead to sleepless nights, thereby reinforcing fatigue” (Cramer et al., 2010, pp. 140–141).

By emphasising the causal relations between the symptoms themselves, the symptom network approach accounts for why the symptoms associated with a given psychiatric diagnosis tend to cluster together in a statistically significant way, without the need to invoke an underlying latent pathology as the cause of these symptoms. Fatigue, poor concentration, worry, and sleepless nights cluster together because they causally reinforce each other, not because they are caused by a common underlying pathology. Hence, by defining a psychiatric disorder at the level of its symptoms rather than at the level of underlying biological causal factors, Borsboom and his colleagues can sidestep the problems of heterogeneity and complexity that affect these underlying causal factors.

Defining the disorder at the level of its symptoms has significant implications for diagnostic explanation. In their commentary on Cramer et al.'s (2010) paper, Hood and Lovett (2010) argue that a logical consequence of excluding underlying causes from the conceptualisation of a psychiatric disorder is that the disorder cannot then function as a causal explanation of a patient's symptoms. If MDD, for example, is nothing over and above the symptoms of low mood, anhedonia, fatigue, and so forth, then to invoke the diagnosis of MDD as an explanation of why these symptoms occur in a particular patient would be tautological. However, even if Hood and Lovett are right in claiming that a disorder cannot be the cause of its symptoms if it is nothing over and above these symptoms, I argue that the symptom network approach enables a psychiatric diagnosis to provide causal information of a different sort. In particular, it provides information about the above mentioned causal relations between the symptoms themselves. It is in virtue of this causal information that the symptom network approach distinguishes between an arbitrary grouping of symptoms and a grouping of symptoms that reflect the causal structure of the world. As argued by Borsboom and Cramer:

In addition, network modeling has the philosophical advantage of dropping the unrealistic idea that symptoms of a single disorder share a single causal background, while it simultaneously avoids the relativistic consequence that disorders are merely labels for an arbitrary set of symptoms … (Borsboom & Cramer, 2013, p.93)

This suggests that although the symptom network model defines a psychiatric diagnosis at the level of its symptoms, the diagnosis does not merely serve as a descriptive label for these symptoms, but also provides additional information about the causal relations that sustain these symptoms as a stable cluster.

Consider the patient who presents to the clinic with low mood, poor concentration, fatigue, and insomnia. According to the symptom network approach, the diagnosis of MDD indicates that these symptoms constitute a dynamically stable system held together by causal relations. Again, this does not meet the standard model of explanation where a diagnosis picks out an underlying pathology that is causing the patient's symptoms, but there is nonetheless good reason to think of it as being a sort of causal explanation. In particular, it explains why the patient's symptoms of occur concomitantly. By positing causal relations between the symptoms, the diagnosis of MDD explains why they have aggregated and persisted as they have, regardless of what pathological processes may be underlying them in the particular case.

Hence, if the symptom network approach is assumed, a psychiatric diagnosis can provide some causal explanatory information about a patient's symptoms, even if the underlying causes of the symptoms vary across cases. However, it is causal explanatory information of a different sort from that provided by a medical diagnosis like MI, which picks out an underlying cause of the patient's chest pain. Again, a claim such as “the patient's mood symptoms are caused by MDD” is misguided, this time because the symptom network model suggests that MDD does not refer to a latent underlying pathology responsible for the symptoms, but we can still claim that the diagnosis of MDD causally explains the patient's symptoms on the grounds that it refers to the causal structure by which the symptoms induce and reinforce each other.

The claim that a psychiatric diagnoses provide information about the causal structures by which sets of symptoms are maintained sits well with the fact that specific therapies for some psychiatric disorders often achieve reductions in some symptoms by optimally intervening on others (Borsboom & Cramer, 2013, p. 98). For example, cognitive-behavioural therapy for MDD employs the notion that thoughts, actions, emotions, and bodily symptoms can all influence one another. The idea is that intervening on the patient's negative thoughts and level of activity through cognitive restructuring and behavioural activation might then lead to improvements in his or her mood and interest level. Therefore, under the symptom network approach, the causal information conveyed by a psychiatric diagnosis can provide a rational basis for therapeutic intervention.

The symptom network approach, then, makes it possible for a psychiatric diagnosis to convey causal explanatory information about a patient's symptoms without specifying an underlying causative pathology. However, a limitation of the approach is that it may turn out not to be applicable to all major psychiatric diagnoses. For instance, it is not obvious why, in the case of schizophrenia, hallucinations and delusions should be causally connected to blunted affect and catatonic behaviour. Similarly, in the case of bipolar disorder, it is not obvious how mania and depression are supposed to causally induce each other. It appears that in these cases we need to appeal to additional causal variables, such as underlying neurobiological processes, in order to make the link between hallucinations and affective blunting, and the link between mania and depression intelligible. Therefore, while there are plausibly some psychiatric diagnoses that provide causal explanatory information about symptoms without needing to invoke information about the underlying processes, it is unlikely that this is the case for all psychiatric diagnoses.

## 4 Concluding remarks

We should take seriously the possibility that many major psychiatric disorders may turn out to exhibit high degrees of causal heterogeneity and complexity. This paper has examined some of the implications of this for the diagnostic explanation in psychiatry. If it turns out that a given diagnostic category subsumes a variety of different underlying causal structures, then this would suggests that diagnostic explanation in psychiatry falls short of the essentialistic ideal where a diagnosis specifies the causative pathology responsible for the patient's symptoms. Nonetheless, I have argued that some psychiatric diagnoses can still provide other sorts of causal information that can be explanatorily relevant. First, in virtue of the exclusion criteria, a psychiatric diagnosis can sometimes provide negative causal information by ruling out other medical causes. Second, in virtue of our scientific knowledge of some of the various causal factors implicated in psychiatric disorders, a diagnosis can provide some probabilistic or disjunctive information about the possible causal processes that might be relevant to the patient, although this information is likely to be vague and partial given our limited scientific understanding of how these various factors come together. Third, in virtue of the causal relations between the symptoms themselves, a psychiatric diagnosis can provide information about why the patient's symptoms occur together and persist as they do.

The high degrees of causal heterogeneity associated with psychiatric diagnoses suggest a need to review the ways in which diagnostic terms are sometimes communicated in clinical psychiatry. For example, because the category MDD is not associated with a specific causative pathology, there are problems with citing MDD as the “cause” of a patient's low mood in the same way as citing MI as the cause of a patient's chest pain. Such a causal claim risks falsely essentialising MDD. As noted in section 1, this essentialisation is not only misleading to patients, but can encourage harmful stigma (Haslam, 2014). Hence, caution is warranted in the way psychiatric diagnoses are represented in the professional discourse of psychiatry. Nonetheless, I have shown how the causal explanatory roles of psychiatric diagnoses might still be useful in clinical practice, even if they do not pick out specific causal essences. The negative causal information can exclude certain avenues for intervention and offer reassurance to patients. The probabilistic information about possible causal processes can occasionally support therapeutic decisions, although it must be conceded that we are far from being able to specify pathways and biomarkers that could allow for powerful interventions. Finally, the information about the causal relations between symptoms can support therapeutic interventions that target particular symptoms to optimally reduce others.

## Footnotes

1 Data from family, twin, and adoption studies indicates a heritability of approximately 37% (Sullivan, Neale, & Kendler, 2000). Moreover, association studies suggest that this heritable component is not attributable to a single gene, but to the combined effect of many genes, each with a small effect size (Shyn & Hamilton, 2010).

2 For example, it has been estimated that up to 50% of cases of MDD are not associated with HPA axis dysregulation (Cowen, 2002, Palazidou, 2012) and it has been suggested that up to two thirds of cases of MDD may not be related to underactive 5-HT neurotransmission (Belmaker & Agam, 2008).

3 Similarly, a review by Roiser et al. (2012) suggests that some patients with MDD have abnormal ACC activation during emotional processing while other patients have normal baseline ACC activation, with the former group showing most improvement with pharmacological treatment and the latter showing most improvement with psychological therapy. While this has potential prospects for treatment selection, it rests on the finding that there is variability of brain mechanisms among patients with the diagnosis of MDD.

4 The variability of brain mechanisms across cases is further highlighted in a meta-analysis by Graham et al. (2013), who find several different brain areas to be associated with MDD, including the occipital cortex, insula, supplementary motor cortex, and the cerebellum, as well as finding contradictory data regarding the activity of the right amygdala. In their conclusion, the authors note that it is not clear that a single neurological model can be applied to all cases of MDD, but disaggregation of clinical subtypes may require different models. There are also other reviews that suggest differences in the neurobiological correlates of different groups of people with MDD (Baumeister and Parker, 2012, Kaufman et al., 2001).

5 Fuchs (2012) argues that psychopathology cannot be understood as being detached from the interpersonal context, because the intrapersonal and interpersonal processes involved are continually intertwined in relations of “horizontal circular causality”. Broome & Bortolotti, (2009) argue that psychiatric symptoms often have intentional content whose meaning is supervenient on the social context in which the patient is situated, and that a description which is exclusively in terms of neurological mechanisms cannot account for the variance in this intentional content.

6 There is evidence to support this. Neuroimaging studies indicate that late-onset MDD is associated with cerebrovascular changes, a greater enlargement in the lateral ventricles, and more white matter hyperintensities, while early-onset MDD is associated with more hippocampal volume loss. Cognitive tests indicate that late-onset MDD, in comparison to early-onset MDD, is associated with decreases in executive function and processing speed (Hermann et al., 2007, Baumeister and Parker, 2012).

7 This is not to say that extensive investigations are always performed whenever a patient presents with mood symptoms. Some conditions may be implicitly excluded due to their unlikelihood in the patient's demographic group, such as a cerebral tumour in a young and otherwise healthy patient with mild depressive symptoms. However, it is normally the case that a patient presenting to secondary care with new affective or psychotic symptoms would at least have blood and urine tests to exclude certain common conditions before a psychiatric diagnosis is established.

8 In this sense, the diagnosis of MDD might be compared to the diagnosis of non-cardiac chest pain, which is a diagnosis that is made when a patient presents with central chest pain but investigations reveal no evidence of cardiac disease. The category does not pick out a specific disease kind, as it encompasses oesophageal, pleuritic, and musculoskeletal pathologies, but it is explanatorily valuable because it excludes cardiac causes of the chest pain.

9 Another example of such a category is cancer. This is highly disjunctive, as it encompasses many different kinds of malignancy. Nonetheless, it is hard to deny that it is of causal explanatory value, as it narrows down possible causal histories, provides some indication of the mechanisms involved in these causal histories, and informs investigations to further specify the diagnosis.
